# Supplementary material for: Adverse drug reactions associated with the use of biological agents
Source: PLoS One. 2020 Dec 18;15(12):e0240276. doi: 10.1371/journal.pone.0240276 (PMC7748267; doi:10.1371/journal.pone.0240276)
Supplement: Attachment — Submitted filename: Review.docx [file pone.0240276.s001.docx]

The article is interesting and brings important information about adverse reactions to biologicals, drugs that are increasingly used. I think it can be accepted for publication after the corrections.

In abstract:

The conclusions item does not answer the objective of the study.

Materials and methods

Line 107-111:

Add the references of the Rawlins and Thompson classification and of ADR probability classification according to the WHO. Put in the table 1 as well.

It would be convenient to explain the Rawlins and Thompson classification (A, B, C, D, E, F) why not all readers know.

Line 112:

"The reported ADRs were standardized according to the WHO adverse reaction terminology (WHO-ART). "Add the references .

The references are not fully in accordance with the journal's rules.
