# Supplementary material for: Adverse drug reactions associated with the use of biological agents
Source: PLoS One. 2020 Dec 18;15(12):e0240276. doi: 10.1371/journal.pone.0240276 (PMC7748267; doi:10.1371/journal.pone.0240276)
Supplement: Attachment — Submitted filename: Reviewer comments ADR Biologics.docx [file pone.0240276.s002.docx]

Reviewer #1: This is a well-conceived study that provides an important assessment of Adverse drug reactions associated with the use of biological agents. This may provide useful information for signal management and benefit-risk assessment.

ABSTRACT

1. Page 2 line 37: Introduction. Biotech drugs open new possibilities to treat diseases for which drug therapy is limited, but they may be associated with serious adverse drug reactions

**Change serious adverse drug reactions to adverse drug reactions in line with your title.**

2. Page 2 line 42-43: The respiratory tract was the most commonly affected organ system (16.8%), followed by the skin and adnexa (15.6%).

**Change adnexa (Latin) to appendages (English) as stipulated by MedDRA.**

**INTRODUCTION**

1. Page 3 line 65-68: Biotech drugs are synthesized from expressed proteins, monoclonal antibodies, vectors (viruses, lipid 66 molecules), antibody fragments, antisense molecules and lipid vectors using innovative genetic engineering methods and recombinant DNA technology, which converts them into drug complexes during manufacturing and these drugs offer the potential to participate in important biological processes in humans [1].

**Sentence is too long. Rewrite sentence.**

2. Page 3 line 68-70**:** Side effects and adverse drug reactions (ADRs) are events that can seriously affect the health of individuals who take drugs for therapeutic, diagnostic or prophylactic purposes.

**I suggest you stick to adverse drug reaction and not side effect based on your topic and objective**

3. **Page 3 line 82:** Because information on the safety associated with the use of biotech drugs, the incidence rates of events and their severity, the causality association and the data on the true benefit/risk ratio are often insufficient, our objective was to identify the ADRs related to the use of biotech drugs in patients affiliated with the Colombian 84 Health System between 2014 and 2019.

**Please note that there is a difference between severity and seriousness according to ICH E2A-Clinical Safety data management: Definitions and standards for expedited reporting. Provide clarification.**

**MATERIALS AND METHODS**

1. Page 4 line 92: The reports are usually made by the treating physicians, nurses responsible for patient care, administrative personnel involved in treatment adherence monitoring or patient support programs and pharmaceutical chemists in charge of pharmacotherapeutic monitoring of ADR reports.

**I believe you are referring here to Pharmacists. Please note that there is a difference between a pharmacist and a pharmaceutical chemist. So, use the appropriate term if you are referring to pharmacists.**

2. Page 4 line 107-114: The general database included the filing date of the ADR report, city, drug (generic name), drug anatomical therapeutic chemical (ATC) classification (letter code and two digits), severity (serious, not serious), type of ADR according to the Rawlins and Thompson classification (A, B, C, D, E, F), ADR probability classification according to the WHO (definitive, probable, possible, unlikely, conditional, unassessable), reported event and the traceability of report submission to INVIMA. The reported ADRs were standardized according to the WHO adverse reaction terminology (WHO-ART). The main drugs for which ADRs were reported were classified, describing the first 15 ATC subgroups (letter code and first two digits), and an ADR list was created for the 10 drugs with the highest numbers of reports.
a. **Provide references and versions for the classifications based on:**
   i. **ATC**
   ii. **WHO-ART**
   iii. **Severity**
   iv. **Causality**
   v. **Types of ADR**
b. **Please note that there is a difference between severity and seriousness according to ICH E2A- Clinical Safety data management: Definitions and standards for expedited reporting. Seriousness is into non-serious and serious and the reasons for seriousness ranges from death, congenital anomaly, disability, hospitalization etc. Severity on the other hand based on the type of scale can be into mild, moderate or severe or grade 1, grade 2 grade 3 etc.**
c. **WHO-UMC system for causality is into certain, probable, possible, unlikely, conditional and unaccessible. Take note and review appropriately**

**RESULTS**

1. Page 5: Table 1. Number of reports per year, classification according to severity and type of adverse reactions in 127 patients treated with biological agents in Colombia 2014-2019
   a. **Your classification based on severity as stated in the table is flawed as I stated previously according to ICH E2A. Clarify whether you are classifying based on severity or seriousness.**
   b. **Under types of adverse reactions, review whether F stands for Failure or Foreign.**
2. Page 7 line 145-146: The causality analysis determined that most ADRs were considered possibly associated with the reported drug (67.9% were definitive, probable or possible) (Figure 1).
a. **Rewrite this sentence and replace definitive with certain according to the WHO-UMC causality assessment system**
b. **Review Figure 1 since the WHO-UMC causality assessment analysis and terms does not take into consideration therapeutic failure and not evaluable**

GENERAL COMMENT:

**This manuscript could benefit from a professional manuscript editing to improve its grammatical, spelling and intellectual clarity.**
